# Assessing the accuracy and consistency of responses from Chat Generative Pre-trained Transformer in human papillomavirus infection and protection

**DOI:** 10.1590/1806-9282.20251769

**Published:** 2026-06-15

**Authors:** Oguz Arslan, Burak Giray

**Affiliations:** 1 Dokuz Eylul University, School of Medicine, Department of Obstetrics and Gynecology, Division of Gynecologic Oncology – Izmir, Turkey.; 2 Koc University, School of Medicine, Department of Obstetrics and Gynecology, Division of Gynecologic Oncology – Istanbul, Turkey.

**Keywords:** Artificial intelligence, ChatGPT, Human papillomavirus, Pap smear, Vaccination

## Abstract

**OBJECTIVE:**

Chat Generative Pre-trained Transformer is an artificial intelligence chat system that is pre-trained on a large dataset of language data, providing detailed answers to user inquiries. The aim of this study was to evaluate the accuracy and consistency of the model's answers to frequently asked patient questions about human papillomavirus infection.

**METHODS:**

The questions prepared for input into Chat Generative Pre-trained Transformer were sourced from globally recognized associations and organizations and were divided into four categories: basic knowledge, diagnosis, treatment, and preventive medicine. The evaluation levels consisted of comprehensive, correct but inadequate, some correct and some incorrect, and completely incorrect. Three independent reviewers evaluated the responses.

**RESULTS:**

A total of 123 questions about human papillomavirus infection were prepared. Of the questions prepared for entry into Chat Generative Pre-trained Transformer related to human papillomavirus infection, 52% were categorized as preventive medicine, 28.5% as basic knowledge, 13.8% as diagnosis, and 5.7% as treatment. The model provided "comprehensive" answers to 115 out of 123 (93.5%) questions. When examined by category, the model provided comprehensive answers to 94.3% of the questions related to "basic knowledge," 88.2% of the questions about "diagnosis," 100% of the questions about "treatment," and 93.8% of the questions about "preventive medicine." Additionally, the model provided reproducible answers to 120 (97.6%) questions.

**CONCLUSION:**

Chat Generative Pre-trained Transformer performed well by providing accurate and consistent answers to frequently asked questions about human papillomavirus infection risk factors, transmission, diagnosis, impacts, associated cancers, prevention methods, and vaccination. The model may be considered a valuable informational resource for patients when used in conjunction with care provided by healthcare professionals.

## INTRODUCTION

Human papillomavirus (HPV) is a common infection primarily transmitted through sexual contact and causes cellular changes in the oral mucosa and genital areas^1^. Nearly 80% of sexually active women get an HPV infection at some point in their lives. The natural immune system responds to and clears approximately 90% of HPV infections, preventing them from becoming persistent^2^. Cervical cancer, arising from HPV infection, is the second most prevalent cancer among women under 50 and the fourth most prevalent across all age groups^1^.

Chat Generative Pre-trained Transformer (ChatGPT) is an artificial intelligence (AI) chatbot that became available to people on November 30, 2022. The technology developed by OpenAI is trained to track user questions and provide detailed responses^3^. In the digital age, access to health-related information has expanded beyond traditional sources like doctors, printed materials, and common search engines. This change in how people look for information is especially notable among younger individuals, who make up a large part of chatbot users and are also more likely to seek information about sexually transmitted infections such as HPV. Issues related to sexual health, including HPV infection, may be perceived as sensitive or stigmatizing, leading some individuals to avoid direct consultation with healthcare providers. In this context, the anonymity and accessibility of AI-based chat systems may make them a preferred initial source of information, as similarly observed in studies on AI-generated responses to musculoskeletal conditions^4^.

In our study, we aimed to evaluate the accuracy, consistency, and reproducibility of ChatGPT's responses to questions that patients may have about HPV infection, including diagnosis, risk factors, transmission, prevention, vaccination, treatment, management of complications, and screening.

## METHODS

A total of 123 questions about HPV infection were prepared to ask ChatGPT. Questions were collected from the frequently asked questions pages of the World Health Organization, Pan American Health Organization, Centers for Disease Control and Prevention, United Nations Children's Fund, and the immunize.org website. Questions with the same meaning, unrelated to HPV infection, and unclear meaning were not included in the study. The questions were edited for grammar and spelling. We considered how patients would ask ChatGPT the questions they wanted to learn the answers to, and we intentionally rephrased the questions like they were written in the first person and revised them into colloquial words. The questions were asked to the model in May and July 2024. The questions were categorized into four categories: basic knowledge, diagnosis, treatment, and preventive medicine, to enable domain-specific evaluation of ChatGPT's performance. This classification approach has been previously used in studies assessing ChatGPT responses in various medical fields, allowing comparison across clinically relevant content areas rather than relying solely on overall accuracy metrics^5–7^. ChatGPT version 3.5 was utilized in the study. ChatGPT 3.5 was preferred due to its free and would be easier to access. The decision to choose the free version in this study was influenced by the fact that HPV infection is more common in low- and middle-income countries and the more significant usage of the free version in these regions. Each question was independently entered into ChatGPT twice at different times, and two separate answers were obtained. This method was aimed at evaluating the consistency of ChatGPT's responses to the questions. The model was prompted to respond using the new chat feature when transitioning from one question to another.

We reviewed ChatGPT's answers for both accuracy and consistency. Answers were evaluated for accuracy in four categories: 1. Comprehensive (the answer is correct, and the gyneco-oncologist has no additional comments to add to ChatGPT's response), 2. Correct but inadequate (the answer is correct but incomplete, and the gyneco-oncologist has information to add to ChatGPT's response.), 3. Some correct and some incorrect, 4. Completely incorrect. To reduce subjectivity in accuracy assessment, predefined criteria were applied. Responses were classified as comprehensive if they were factually correct, addressed all key clinical and educational aspects of the question, and required no additional clarification or supplementation by a gyneco-oncologist. Responses were classified as correct but inadequate if they were factually accurate but incomplete, lacking one or more essential elements that would be included in standard patient counseling, such as clarification of risks, limitations, indications, contraindications, or contextual explanations. Given that ChatGPT version 3.5 is limited by a January 2022 knowledge cutoff, this temporal constraint was taken into consideration during the evaluation of its responses. We evaluated ChatGPT's two answers to each question to analyze consistency. Two groups were formed to evaluate the answers: Group 1: Categories 1 and 2, Group 2: Categories 3 and 4. At the end of the scoring process, answers assigned to the same group were considered consistent and reproducible, reflecting rating-level reproducibility rather than semantic equivalence of the response content. Answers were independently evaluated for accuracy and consistency by gyneco-oncologist (OA and BG). At the end of the evaluation, the two reviewers accepted answers if they were consistent across evaluation categories and groups. In cases of disagreement between the two primary reviewers, the response was reviewed by a third board-certified senior gynecologic oncologist (MA) with 39 years of experience in gynecologic oncology, who facilitated a consensus decision. The third reviewer did not independently reassess all responses but was involved only in resolving discordant evaluations. The three reviewers were independent and blind to each other when assessing the questions. Accuracy assessments were informed by current evidence-based clinical guidelines and standard practice recommendations.

Ethics committee approval was not received due to the nature of the study and the fact that the answers provided by ChatGPT were public. Data was analyzed by Statistical Package for the Social Sciences (Version 21.0, IBM SPSS Statistics for Windows; IBM Corp. Armonk, NY, USA). Frequency and ratio values were used for descriptive statistics. The questions were evaluated both within the category and overall. A Kappa analysis was performed to assess the agreement between the two reviewers.

## RESULTS

Of the 123 questions prepared for entry into ChatGPT related to HPV infection, 52% were categorized as preventive medicine, 28.5% as basic knowledge, 13.8% as diagnosis, and 5.7% as treatment. Only a subset of representative questions is shown in Table 1.

**Table 1:** Questions entered into Chat Generative Pre-trained Transformer by category.

Basic knowledge n=35 (28.5)	Preventive medicine n=64 (52)
What is human papillomavirus (HPV)? How can HPV be transmitted? Is sexual contact the only way of getting HPV? How long does HPV infection last? Does HPV cause cancer? What types of HPV infection cause cancer? Can all HPV types cause cervical cancer?	What is the HPV vaccine? When did the HPV vaccination program start? Can HPV infection be prevented? How can you protect against HPV infection? How are HPV infection and cervical pre-cancer screened for? How can HPV and cervical cancer be prevented? What vaccines are available to prevent HPV? Who recommends the HPV vaccine? What are the HPV vaccines composed of? When should people get the HPV vaccine? How effective is the HPV vaccine? When were the HPV vaccines licensed? How is this HPV vaccine given? What protection is provided by one HPV vaccine dose? Who should get the HPV vaccine? Should individuals be screened before getting HPV vaccinated? Why does my child need HPV vaccination? How long does HPV vaccine protection last? What is the recommended schedule for HPV vaccines? Are the HPV vaccines productive and effective in preventing cervical cancer? Can the HPV vaccine be co-administered with other vaccines? Do the children need consent from their parents or caregivers to be HPV vaccinated? Does the vaccine protect against all types of HPV?
Diagnosis n=17 (13.8)	
How is HPV infection diagnosed? Why test for HPV? If my HPV test was positive, what can I tell my partner? Can I ask for an HPV test at any time? If I have HPV, can I ask for my partner to have a test too? Can I get the same type of HPV more than once? How do we test for high-risk HPV?	
Treatment n=7 (5.7)	
Can HPV infection be treated? How are genital warts treated? Is there an immune response after a natural HPV infection? What does the "screen-and-treat" approach mean in HPV? How are HPV infection and cervical pre-cancer treated? As I get older, will I be less able to get rid of HPV? In the past, I had cervical cell changes that went away by themselves. Does this mean my immune system is now strong enough to fight HPV?	

Values are presented n (%). HPV: human papillomavirus.

The model provided "comprehensive" answers to 93.5% of 123 questions, "correct but inadequate" answers to 4.1%, and "some correct, some incorrect" answers to 2.4%. At the end of the evaluation of all answers, no response was detected as "completely incorrect." The distribution of comprehensive answers by category is 100% in the treatment category, followed by basic knowledge at 94.3%, preventive medicine at 93.8%, and diagnosis at 88.2%. The rate of "correct but inadequate" responses was 5.7 and 4.7% in the basic knowledge and preventive medicine categories, respectively, with no responses as correct but inadequate in the diagnosis and treatment categories. The rate of "some correct, some incorrect" responses was 11.8% in the diagnosis category, 1.6% in the preventive medicine category, and none in the basic knowledge and treatment categories (Table 2).

**Table 2:** Grading of responses generated by Chat Generative Pre-trained Transformer to questions related to human papillomavirus infection categorized by question type.

Basic knowledge (n=35)	
Comprehensive	33 (94.3)
Correct but inadequate	2 (5.7)
Some correct and some incorrect	0 (0)
Completely incorrect	0 (0)
Diagnosis (n=17)	
Comprehensive	15 (88.2)
Correct but inadequate	0 (0)
Some correct and some incorrect	2 (11.8)
Completely incorrect	0 (0)
Treatment (n=7)	
Comprehensive	7 (100)
Correct but inadequate	0 (0)
Some correct and some incorrect	0 (0)
Completely incorrect	0 (0)
Preventive medicine (n=64)	
Comprehensive	60 (93.8)
Correct but inadequate	3 (4.7)
Some correct and some incorrect	1 (1.6)
Completely incorrect	0 (0)
Total (n=123)	
Comprehensive	115 (93.5)
Correct but inadequate	5 (4.1)
Some correct and some incorrect	3 (2.4)
Completely incorrect	0 (0)

Values are presented n (%).

ChatGPT provided reproducible answers to 120 (97.6%) of the questions. The distribution of reproducible responses by category is as follows: treatment 100%, preventive medicine 98.4%, basic knowledge 97.1%, and diagnosis 94.1% (Table 3).

**Table 3:** The proportion of questions with reproducible responses generated by Chat Generative Pre-trained Transformer.

Category of the questions	Reproducible responses
Basic knowledge (n=35)	34 (97.1)
Diagnosis (n=17)	16 (94.1)
Treatment (n=7)	7 (100)
Preventive medicine (n=64)	63 (98.4)
Total (n=123)	120 (97.6)

Values are presented n (%).

Reviewers 1 and 2 evaluated ChatGPT's answers and determined that 95 (77.2%) questions were compatible. After the two reviewers’ evaluation, the third reviewer evaluated and resolved 28 (22.8%) incompatible questions. In the kappa analysis conducted to test the reliability between the first two reviewers, the kappa value was 0.746, indicating a substantial level of agreement between them^8^.

## DISCUSSION

Since its launch, ChatGPT has continued to grow in popularity, consistently surprising people with its capabilities. In this study, 52% of the questions submitted to the model were related to preventive medicine. Due to the nature of publicly available HPV-related educational materials, questions focusing on preventive medicine, particularly those addressing vaccination and protection, were more frequently represented in the question set. This distribution likely reflects real-world patient information-seeking behavior, in which prevention-oriented topics constitute a substantial proportion of publicly accessible health information. Our study found that the model's answers to questions about HPV infection were 93.5% comprehensive, 97.6% reproducible, and consistent. In the study by Samaan et al., which evaluated ChatGPT's answers to questions about bariatric surgery, it was shown that the model provided responses that were 86.8% comprehensive and 90.7% reproducible^5^. In Yeo et al.'s study assessing the performance of ChatGPT, the model's comprehensive and correct response rates to questions about cirrhosis were 47.3 and 79.1%, and its comprehensive and correct response rates to questions about hepatocellular cancer were 41.1 and 74%, respectively^6^. In the study by Hermann et al., which analyzed ChatGPT's accuracy regarding cervical cancer, the comprehensiveness rate of the model's answers was determined to be 53.1%^7^. As shown, some results from studies in the current literature that assess ChatGPT's knowledge of medical subjects are consistent with our findings, but some are not. We believe that this difference arises for two reasons. The first reason is that the studies investigate different areas of medicine and various medical subjects. In our study, we selected HPV infection, a common condition that affects all sexually active men and women in the population. In line with this choice, the questions we prepared for ChatGPT focused primarily on topics of broad public concern related to HPV infection, such as transmission, screening, prevention, and vaccination, rather than on technical or exam-related medical information. Cirrhosis, hepatocellular cancer, and bariatric surgery are health problems that affect only a portion of the population and are not as common and frequent as HPV infection. The second reason, we believe, arises from differences in the number of questions used in the studies. The number of questions in our study and Samaan's study was 123 and 151, respectively. Hermann et al. used 64 questions in their study, nearly half the number used in our study. Increasing the number of questions used in studies will allow for a better evaluation of ChatGPT's performance and result in stronger statistical analyses.

HPV is highly prevalent, with reports indicating that 84.6% of women and 91.3% of men will be infected at some point in their lives^9^. A study conducted in the United States reported an HPV prevalence of 40%^10^. Although HPV is very common, public health literacy about it is not at the desired level. Studies have reported that increased health literacy regarding HPV leads to higher rates of Pap smear screening and vaccination for protection against infection^11,12^. The tailored solutions developed by policymakers to improve access to accurate health-related information and health literacy do not reach the entire public promptly and effectively. In some regions, patients have difficulty accessing healthcare providers^13^. We believe that ChatGPT can help bridge this gap. The model will help alleviate patients’ concerns at the initial step with its performance in both general and sub-domains (basic knowledge, diagnosis, treatment, and prevention medicine) related to HPV infection. Patients can benefit from AI technology that communicates with them conversationally, as an informative resource. The accessibility of the free version of ChatGPT v3.5 is an important factor contributing to its increased use. We believe that increasing awareness of HPV, particularly among individuals in countries with low socioeconomic development, will positively impact screening and vaccination rates.

Platforms with online access, particularly popular free social media resources such as YouTube and Twitter, are widely used by patients worldwide as sources of health information^14^. Patients seeking information about HPV infection, which ranks first among sexually transmitted diseases, also refer to social media content. The accuracy and reliability of content produced in digital media are unclear. The results of studies examining social media content regarding HPV infection are insufficient and inconsistent with each other. While health professionals share information on these platforms, individuals with ulterior motives aim to spread false and harmful information. It is easy to access both accurate and inaccurate information, and patients may struggle to assess the accuracy of the information. The spread of misinformation to the public and the rise in false beliefs about HPV infection may complicate the course and management of the disease^15,16^. Since patients cannot discern the accuracy of HPV-related content on social media, ChatGPT could help address this challenge. The advantages of ChatGPT include its high accuracy and repeatability in responses and its use of language that is more comprehensible to patients. Additionally, the model adjusts its responses based on user feedback. We believe that ChatGPT can potentially reduce inequalities in access to health information owing to its advanced features. However, the use of AI-based tools, such as ChatGPT, in healthcare also raises important ethical and medicolegal issues. These include liability for incorrect information, data privacy concerns, and the risk that patients, especially in low-resource settings, may rely on AI-generated advice instead of professional medical care. Therefore, such tools should be implemented with clear safeguards, transparency regarding their limitations, and explicit emphasis on their role as supportive rather than substitutive resources within the healthcare system.

Although ChatGPT possesses impressive features, it can also produce false and inaccurate information. In our study, 2.4% of the model's responses fall into the "some correct, some incorrect" category. Other studies indicate that the model produces incorrect information^6,7^. We believe that the basis of the misinformation generated by the model may be attributed to the lack of transparency regarding the sources of information used in its development, its ongoing development, and the delay in updating with current literature. According to a statement from OpenAI, the model is expected to improve and update its information based on the feedback it receives^17^. Large language models, including ChatGPT, have been shown to generate plausible but incorrect or misleading information, commonly referred to as hallucinations^18^. Although only 2.4% of responses in our study were classified as partially incorrect, even low rates of misinformation may pose clinically meaningful risks in healthcare settings. These findings underscore the importance of expert oversight and emphasize that AI-generated health information should not be used without a suitable clinical context.

This study is the first to evaluate ChatGPT's accuracy and consistency regarding HPV, with independent expert review enhancing its robustness. Given the global prevalence of HPV, the findings have broad applicability. Although response evaluations were informed by current clinical guidelines and expert knowledge, the study did not include a structured, item-level comparison with specific guideline documents. Another limitation of this study is the exclusive evaluation of ChatGPT version 3.5. Although newer versions, such as ChatGPT-4, have demonstrated superior performance, their restricted accessibility may limit real-world use in certain populations. The small number of questions in the treatment category limits the interpretability and generalizability of this finding. no inferential statistical tests or adjustments for multiple comparisons were performed, and category-based differences are therefore descriptive rather than statistically supported. Another limitation of this study is the absence of systematic prompt engineering. This approach was chosen to reflect real-world patient use, in which questions are typically asked in informal, non-optimized language. ChatGPT may produce inaccuracies, and healthcare practices vary; it should serve only as a supplementary resource, with final decisions left to healthcare professionals.

## CONCLUSION

Patients often have difficulty accessing reliable information about HPV, as current information sources are limited and unreliable. ChatGPT may positively contribute to the increase in health literacy. These findings are limited to expert-reviewed responses and do not address patient interpretation, behavioral impact, or clinical outcomes. The model may be considered a valuable informational resource for patients when used in conjunction with care provided by healthcare professionals; however, it should be regarded solely as a supplementary tool and should not replace professional medical consultation.

## Data Availability

The datasets generated and/or analyzed during the current study are available from the corresponding author upon reasonable request.
